# P008 Bilateral Stress Fractures Revealing FGF23-Mediated Hypophosphatemia Rickets in an Adult with Co-existing Endocrine Disorders

**DOI:** 10.1093/rap/rkag113.038

**Published:** 2026-11-03

**Authors:** Huma Liaqat, David Collins

**Affiliations:** Great Western Hospital NHS Trust, Swindon, United Kingdom; Great Western Hospital NHS Trust, Swindon, United Kingdom

## Abstract

**Introduction:**

Atraumatic bilateral stress fractures in adults should prompt investigation for underlying metabolic bone disease. Hypophosphatemia rickets is a rare but important cause of osteomalacia, often presenting with non-specific musculoskeletal symptoms and delayed diagnosis. Co-existing endocrine abnormalities may further obscure recognition. We present a diagnostically challenging case of progressive insufficiency fractures initially attributed to vitamin D deficiency and hypothyroidism, in whom persistent biochemical abnormalities ultimately led to the diagnosis of FGF23-mediated hypophosphatemia rickets.

**Case description:**

A 49-year-old woman with longstanding hypothyroidism presented with progressive atraumatic bone pain and worsening mobility. Initial imaging demonstrated bilateral stress fractures involving the medial cortices of the proximal femora, consistent with Looser zones. Over subsequent months, she developed widespread skeletal pain involving the ribs, sacrum and feet, with further imaging confirming multifocal insufficiency fractures.

Biochemical assessment revealed persistent hypophosphatemia (0.45–0.54 mmol/L), low-normal calcium and elevated alkaline phosphatase (>200 IU/L). Vitamin D deficiency was initially identified (25-hydroxyvitamin D 38 nmol/L; reference >50 nmol/L) and treated with supplementation. Despite subsequent improvement in vitamin D levels, symptoms and biochemical abnormalities persisted. Early management focused on presumed osteomalacia secondary to vitamin D deficiency and undertreated hypothyroidism, with optimization of levothyroxine and vitamin D replacement.

Further investigation demonstrated inappropriate renal phosphate wasting, supported by elevated urinary phosphate excretion and a reduced tubular maximum phosphate reabsorption rate (TmP/GFR) of 0.36. Serum FGF23 was elevated at 186 (reference <100), confirming FGF23-mediated hypophosphataemia. Genetic testing for recognised hereditary causes was negative, suggesting a sporadic form.

Treatment with oral phosphate supplementation and calcitriol was initiated and gradually titrated because of gastrointestinal side effects. This resulted in biochemical improvement, radiological fracture healing and substantial functional recovery from severe mobility limitation to independent ambulation, although mild hypophosphatemia persisted.

**Discussion:**

This case highlights the diagnostic complexity of adult osteomalacia and underscores the importance of recognizing persistent hypophosphatemia as a significant diagnostic clue. Bilateral femoral stress fractures and Looser zones are classical manifestations of impaired bone mineralization and should immediately prompt consideration of an underlying metabolic bone disorder. Although uncommon, FGF23-mediated hypophosphatemia represents an important and potentially treatable cause of osteomalacia in adults and may be overlooked because of its non-specific clinical presentation.

The coexistence of hypothyroidism and vitamin D deficiency initially appeared to provide an adequate explanation for the patient’s symptoms, biochemical abnormalities and fractures. However, despite optimization of thyroid replacement and correction of vitamin D deficiency, the patient remained symptomatic and persistently hypophosphatemia. The prolonged persistence of hypophosphatemia despite vitamin D repletion represented the key diagnostic clue that prompted further investigation beyond more common endocrine causes of osteomalacia.

Assessment of renal phosphate handling proved pivotal in establishing the diagnosis. Demonstration of inappropriate phosphate wasting, supported by elevated urinary phosphate excretion and a markedly reduced TmP/GFR, suggested an alternative pathogenic mechanism. Subsequent identification of elevated FGF23 confirmed an FGF23-mediated disorder. The absence of a relevant family history and negative genetic testing favored a sporadic or acquired form of hypophosphatemia rickets.

Delayed diagnosis of FGF23-mediated hypophosphatemia can result in prolonged disability, recurrent insufficiency fractures and substantial reductions in quality of life. This case demonstrates the value of a structured approach to persistent hypophosphatemia, including assessment of urinary phosphate excretion, calculation of TmP/GFR and timely measurement of FGF23. Early recognition is essential, as targeted treatment with phosphate supplementation and calcitriol can promote fracture healing, improve biochemical abnormalities and restore physical function.

**Key learning points:**

• Bilateral or atraumatic stress fractures, particularly in the presence of Looser zones, should prompt urgent evaluation for underlying metabolic bone disease.

• Persistent hypophosphatemia is an important diagnostic clue and should not be attributed solely to vitamin D deficiency or other co-existing endocrine disorders.

• Assessment of renal phosphate handling, including urinary phosphate excretion and TmP/GFR, is essential when investigating unexplained hypophosphatemia.

• Reduced TmP/GFR is a key indicator of inappropriate renal phosphate wasting.

• Measurement of FGF23 is critical for identifying FGF23-mediated hypophosphatemia disorders and guiding diagnosis.

• Adult-onset FGF23-mediated hypophosphatemia may occur sporadically in the absence of a family history or identifiable genetic mutation.

• Delayed recognition can result in recurrent insufficiency fractures, prolonged morbidity and significant functional impairment.

• Early targeted treatment with phosphate supplementation and calcitriol can improve biochemical abnormalities, promote fracture healing and restore function.

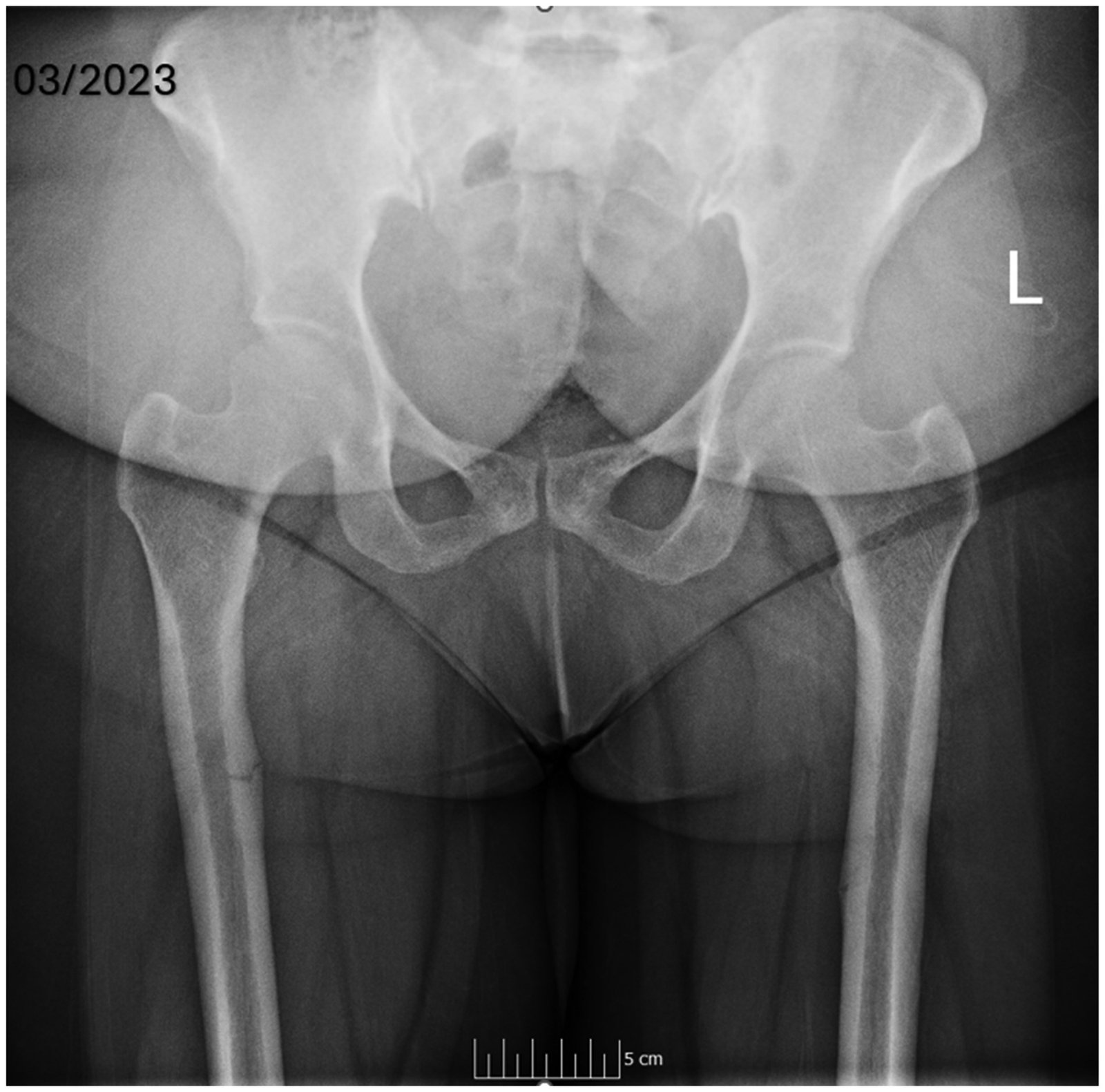

**P008 Figure 1**

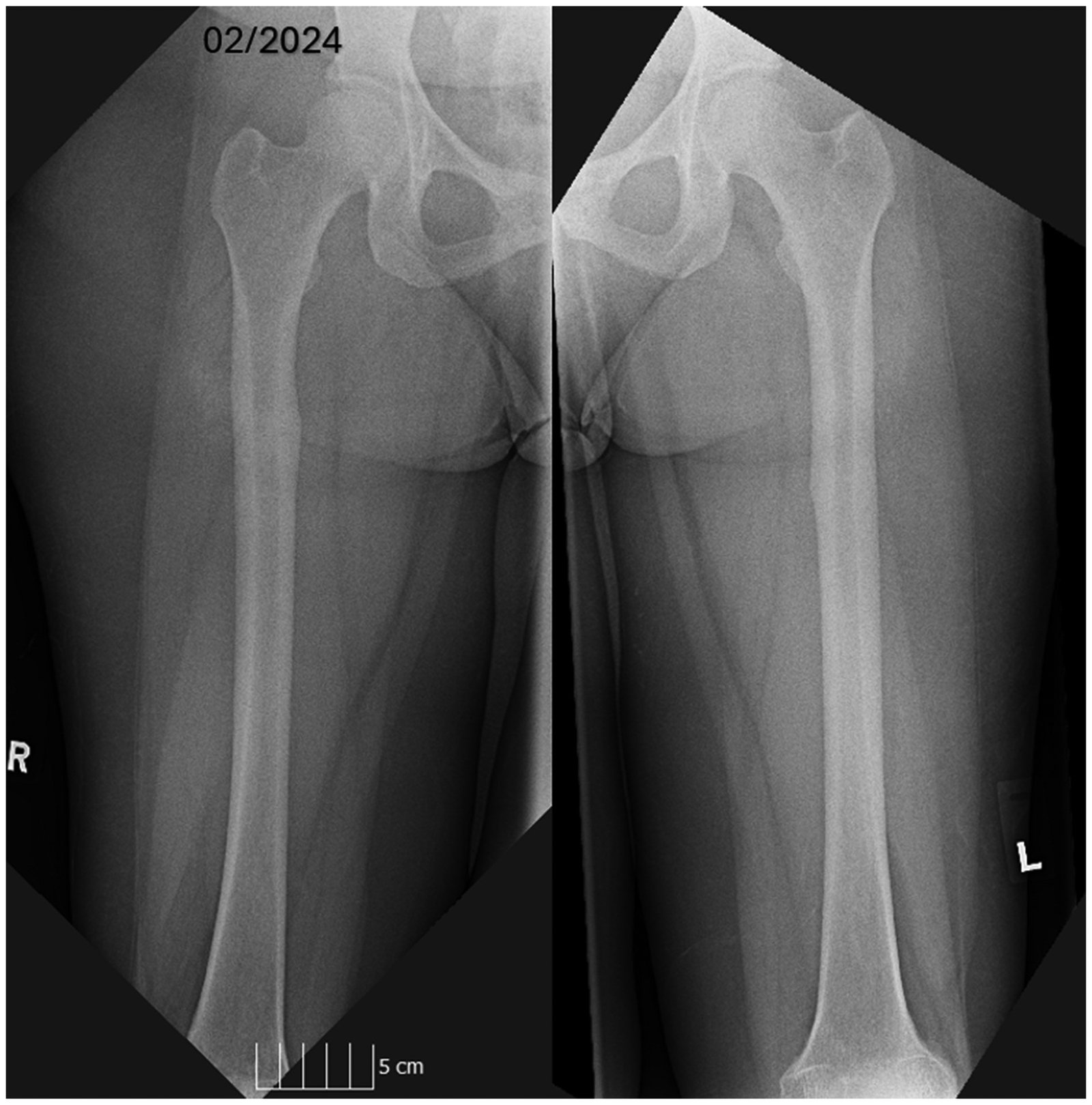

**P008 Figure 2**

**P008 Table 1:** 

Investigations	Result
Phosphate	0.45–0.54 mmol/L
ALP	>200 IU/L
Vitamin D	38 → 64 → 82 → 73 → 84 → 105 → 47 → 57 nmol/L
Urine phosphate	16.40 mmol/L
TmP/GFR	0.36
FGF23	186 (<100)

